# Patient Survival and Costs on Moderately Restricted Low-Protein Diets in Advanced CKD: Equivalent Survival at Lower Costs?

**DOI:** 10.3390/nu8120758

**Published:** 2016-11-25

**Authors:** Giorgina Barbara Piccoli, Marta Nazha, Irene Capizzi, Federica Neve Vigotti, Elena Mongilardi, Marilisa Bilocati, Paolo Avagnina, Elisabetta Versino

**Affiliations:** 1 Department of Clinical and Biological Sciences, University of Torino, Torino 10100, Italy; marta.nazha@gmail.com (M.N.); irene.capizzi@gmail.com (I.C.); fesesnow@inwind.it (F.N.V.); elenamongilardi@yahoo.it (E.M.); 2 Nephrologie, Centre Hospitalier Le Mans, Le Mans 72000, France; 3 Obstetrics, Department of Surgery, Città Della Salute e Della Scienza, University of Torino, Torino 10100, Italy; marilisa.biolcati@unito.it; 4 SSD Clinical Nutrition, Department of Clinical and Biological Sciences, ASOU san Luigi, University of Torino, Torino 10100, Italy; paolo.avagnina@unito.it; 5 SS Epidemiology, Department of Clinical and Biological Sciences, ASOU san Luigi, University of Torino, Torino 10100, Italy; elisabetta.versino@unito.it

**Keywords:** low protein diet, dialysis start, mortality, standardized mortality ratio, registry, costs

## Abstract

The indications for delaying the start of dialysis have revived interest in low-protein diets (LPDs). In this observational prospective study, we enrolled all patients with chronic kidney disease (CKD) who followed a moderately restricted LPD in 2007–2015 in a nephrology unit in Italy: 449 patients, 847 years of observation. At the start of the diet, the median glomerular filtration rate (GFR) was 20 mL/min, the median age was 70, the median Charlson Index was 7. Standardized mortality rates for the “on-diet” population were significantly lower than for patients on dialysis (United States Renal Data System (USRDS): 0.44 (0.36–0.54); Italian Dialysis Registry: 0.73 (0.59–0.88); French Dialysis Registry 0.70 (0.57–0.85)). Considering only the follow-up at low GFR (≤15 mL/min), survival remained significantly higher than in the USRDS, and was equivalent to the Italian and French registries, with an advantage in younger patients. Below the e-GFR of 15 mL/min, 50% of the patients reached a dialysis-free follow-up of ≥2 years; 25% have been dialysis-free for five years. Considering an average yearly cost of about 50,000 Euros for dialysis and 1200 Euros for the diet, and different hypotheses of “spared” dialysis years, treating 100 patients on a moderately restricted LPD would allow saving one to four million Euros. Therefore, our study suggests that in patients with advanced CKD, moderately restricted LPDs may allow prolonging dialysis-free follow-up with comparable survival to dialysis at a lower cost.

## 1. Introduction

Since the beginning of “renal medicine”, low-protein diets (LPDs) have been adopted to correct the metabolic alterations of kidney failure, sometimes interpreted as “protein intoxication” [1,2,3]. To date, the limited availability of dialysis in developing countries and the grim prognosis of patients who start dialysis in developed countries make prolonging dialysis-free survival a major achievement for developed as well as developing countries [4,5,6,7,8,9,10,11,12].

Despite evidence-based studies, including a Cochrane review, suggesting that LPDs delay the need to start renal replacement therapy (RRT), LPDs are still underutilized for several reasons: the time-consuming, educational approach needed to achieve compliance; concerns about malnutrition and impaired survival and of a carry-over effect increasing the risk of death in patients who were previously on a diet [4,5,13,14,15,16,17,18].

Two well-designed randomized trials published in the new millennium dealt with “very low protein diets” (vLPDs: protein intake of 0.3 g/kg of body weight/day), and demonstrated a significant advantage in prolonging dialysis-free survival by means of these demanding dietary options [19,20]. Less is known about the effect of moderately restricted LPDs (protein intake 0.6 g/kg/day), which are feasible in the large majority of chronic kidney disease (CKD) patients, as previously described by several Italian groups, including ours, but are probably the most widely used all over the world [21,22,23,24]. The availability of several diet options, which is the case in the setting of the study, widens the potential of adapting dietary treatments to the needs and preferences of different types of patients [25,26].

In this context, the present study was undertaken to assess patient survival, dialysis-free follow-up and costs of moderately restricted LPDs prescribed with a personalized multiple-choice approach, in which the patients were able to choose among several diet options, described elsewhere in detail [21,22,25]. The study enrolled 449 consecutive patients (with 847 overall patient-years of observation during the diet, and 941 patient-years considering also the first year after the start of dialysis or diet discontinuation), probably representing the largest observational cohort on moderately restricted LPDs available so far.

## 2. Methods

### 2.1. Setting of Study

The study was carried out at the out-patient Nephrology Unit of the san Luigi Hospital, University of Torino, Italy where a moderately restricted low protein diet was routinely proposed by nephrologists to all patients with CKD stages IV–V, or with rapid progression of CKD stage III and/or refractory nephrotic syndrome, in the absence of contraindications (including baseline malnutrition, very short life expectancy, age over 80, in particular in the setting of a stable kidney function) [21,25].

All adult subjects with at least one month of follow-up on a LPD in 2007–2015 were included in the present study. Pregnant patients, who are also prescribed an adapted LPD, as elsewhere described, are not included in the present analysis [27].

### 2.2. Diet and Control Protocols

Moderately restricted LPDs were defined as with a protein intake of 0.6 g/kg/day; patients could choose between a vegan-vegetarian diet supplemented with alpha-ketoacids and amino acids (LPD-AK); a diet with protein-free commercial food (LPD-PFF); different combinations of the previous diets and vegan non-supplemented diets; a very-low protein diet (with a protein intake of 0.3 g/kg/day) integrating LPD-AK and LPD-PFF.

All diets entail a qualitative prescription, based on allowed and forbidden foods and one to three unrestricted meals per week, as described in detail elsewhere [22].

Daily energy intake (aimed at 30–35 kcal/kg/day), supplementation with calcium, vitamin D, folic acid, vitamin B12, iron and erythropoietin followed the usual indications. The frequency of biochemical exams and routine visits was tailored to needs and functional status; overall one clinical visit with biochemical tests was performed each one to three months, according to stage, proteinuria and progression trend; more frequent controls were planned in the case of rapid progression, or at GFR below 10 mL/min [21,22,25].

CKD stages were defined according to the National Kidney Foundation guidelines; e-GFR was calculated with the Chronic Kidney Disease Epidemiology Collaboration (CKD-EPI) formula; compliance as per the Maroni Mitch formula; and comorbidity was weighted with the Charlson Index [28,29,30,31].

Nutritional status was clinically controlled at each visit, taking into account the general clinical status, weight, hydration, blood pressure, the presence of edema, handgrip strength; albumin, hemoglobin, serum electrolytes and total cholesterol were included in the basic tests performed before each clinical control, and bio-impedance was performed on demand, with particular attention to the decrease in lean body mass from baseline; brain natriuretic peptide integrated the work-up in case of over-hydration.

Dialysis start was decided on the basis of the clinical picture, within a policy of “intent to delay”, following the recent guidelines; indications for dialysis were all the uremic signs and symptoms, including uncontrolled hypertension, fluid overload, gastrointestinal symptoms, unexplained fatigue, low hemoglobin levels non responding to optimization of iron stores and erythropoietin, restless leg syndrome and other neurological problems, severe calcium phosphate and parathyroid hormone (PTH) derangements [8,21,22,32,33].

Compliance was routinely assessed on the basis of urinary urea excretion, according to the Maroni Mitch formula [21,22].

### 2.3. Standardized Mortality Rates

Standardized mortality rates were calculated and compared to the most recently published data in the Italian Dialysis Registry (incident patients in 2000–2008); the French Dialysis Registry (mortality rates, prevalent patients 2012; report 2014; data inferred from figures); and the United States Renal Data System (USRDS report 2014: White non-Hispanic patients; all cause mortality by age group (unadjusted); mortality rates 2012) [34,35,36]. The interpretation and reporting of this data are the responsibility of the authors and in no way should be seen as an official policy or interpretation of the U.S. government [34].

We chose not to consider incident patients in the first ESRD period, albeit aware that this choice may underestimate the advantages of LPDs.

The analysis was performed considering overall follow-up on the diet, or limited to the follow-up period after the first recording of an e-GFR ≤15, or ≤10 mL/min. The first year after discontinuation or dialysis start was included in a separate analysis, to control for an eventual negative carry-over diet effect.

### 2.4. Cost Analysis

In the setting of study, both protein-free food and amino and keto-acid supplements are provided free of charge to all CKD patients; the reimbursement for protein free food varies in the different Italian regions. In Piemonte, region of study, the allowance was up to 120 Euros per month, thus a maximum expenditure for protein-free food: 120 Euros per month was calculated for the present analysis. We also considered a cost of 100 Euros per month for the amino and keto-acid supplements (Alfa-Kappa one pill each 10 kg of body weight (BW) in moderately restricted LPDs).

No extra expense was entailed in vegan non-supplemented diets; the cost of diets at 0.3 g/kg IBW/day (v-LPDs) was calculated as 360 Euros per month (Alfa-Kappa 1 pill each 5 kg of body weight, plus protein free food). Balancing the low prevalence of v-LPDs, and the presence of non-supplemented diets, or of personalized diets, which employ lower supplements or commercial food, we estimated a mean cost per year of diet as 1200 Euros.

To calculate cost of dialysis, we considered the recently published average European cost of 50,000 Euros, choosing the lowest figure given, so we would not overestimate the effect of the diet [37].

As for dialysis “exempt” years, we considered the follow-up after the first finding of an e-GFR at or below 15 or 10 mL/min, according to different hypotheses, from 50% of the observed follow-up after e-GFR at or below 10 mL/min (“late” dialysis start) to the overall follow-up at or below 15 mL/min (“early” dialysis start).

Due to the wide diffusion of LPDs in Italy, dietary management is usually a part of the clinical knowledge of the nephrologists. In such a context, the “start-up” time, for setting up an outpatient unit with attention to dietary treatment, could not be calculated.

### 2.5. Statistical Analysis

A descriptive analysis was performed as appropriate (median and range for non-parametric data, mean and standard deviation for parametric distribution), *t*-test was performed according to standard indications for continuous variables, while risks, rates and proportions were compared using Chi-square and Fisher’s exact test. Survival analysis was performed according to Kaplan Meier. Significance of the differences was tested by the log-rank test and Wilcoxon test. The alpha value (statistical significance) was set at 0.05.

### 2.6. Ethical Issues

Informed consent was obtained for anonymous management of the clinical data.

The observational study design (PROTEREne: PROTEin REductio to protect the REins) based on standard clinical practice, that includes patients with severe CKD, was approved by the Ethics Committee of the San Luigi Hospital, University of Torino (Delibera 22, 18 January 2013, protocol 000037).

The database is built as a work in progress, being continuously updated, and data collection will be closed at December 2016 (as for dialysis start and mortality); the data are available upon request to the corresponding author, and will be deposited in a data depository after completion of the final updating.

## 3. Results

### 3.1. Baseline Data

Table 1 reports the main characteristics of the study cohort: the high median age (70 years) and high comorbidity (Charlson Index 7) are comparable to those of patients starting dialysis in our setting; more than half of the patients are affected by diabetes or nephroangiosclerosis or by a combination of the above.

The diet options were almost evenly divided between vegan-supplemented diets (215 patients, 376 years of on-diet observation) and protein-free food (159 patients, 336 years of on-diet observation), while other options, which were progressively developed, accounted for a minority of cases (75 patients, 136 years of on-diet observation) (Table 2). Compliance with protein restriction was good, and without significant difference according to the chosen diet; at the update in March 2016, in 147 on-diet patients, with complete data in the same laboratory, the median protein intake calculated according to the Mitch formula, at least one day after an unrestricted meal, was 0.47 g/kg/day, versus a prescribed intake of 0.6 g/kg/day, as described in detail elsewhere [38,39].

### 3.2. Survival Analysis

Over the 847 years of on-diet follow-up, 100 patients died (22.3%), and 105 started dialysis (23.4%); only eight patients discontinued the LPDs (1.8%); five (1.1%) were lost to follow-up and six (1.3%) were transferred to another center.

As expected, patients who died were older (median age 74 years) and had higher comorbidity (Charlson Index 9) compared to those who started dialysis (median age: 64 years; Charlson Index: 6) (*p* = 0.0001). Nine further deaths were recorded in the first year after discontinuation of the diet or start of dialysis (a further 94.4 years of observation).

The relative risk for mortality (RR) in the on-diet cohort was significantly lower compared to the dialysis populations (USRDS: RR: 0.44 (CI 0.36–0.54); Italian Dialysis Registry: RR 0.73 (CI 0.59–0.88); French Dialysis Registry: RR: 0.70 (CI 0.57–0.85)), with no difference when the first year of follow-up after dialysis start was also considered (Table 2).

When analyzing only the follow-up after the first recording of an e-GFR at or below 15 or 10 mL/min (the “diet or dialysis phase”), the survival advantage was maintained in younger patients (age < 65 years), while mortality rates for older patients were equivalent to those recorded in the two European Registries, and lower than those in the USRDS (Table 3).

### 3.3. Cost Analysis

The analysis was focused on the “diet or dialysis” period, overall encompassing 384 years of follow-up observed after the first observation of e-GFR < 15 mL/min, the conventional limit for “early dialysis start”.

**Figure 1:**
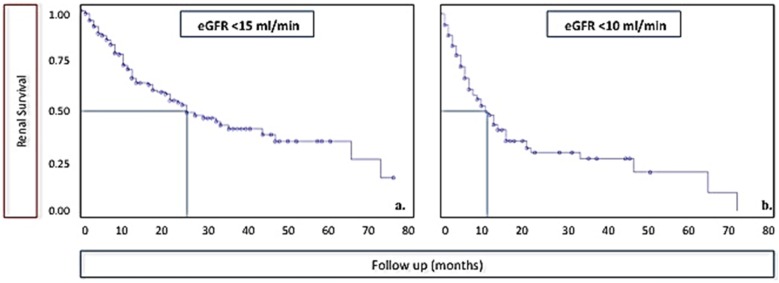
Dialysis-free follow-up after the first finding of an e-GFR at or below 15 mL/min and 10 mL/min.

As shown in Figure 1, half of the patients were able to continue a dialysis-free follow-up for at least two years, 25% for about four. About half of the patients who reached an e-GFR < 10 mL/min were dialysis-free one year later.

Considering an e-GFR of 15 mL/min or less as a marker of “early” dialysis start, and an e-GFR at or below 10 mL/min as a marker of “late” dialysis start, we tried to translate these figures into savings related to the dialysis-“exempt” year: the overall cost of the diet for the 847 years of observation (in 450 patients) was calculated as 1,016,400 Euros, which may also be rounded to 240,000 Euros for 100 patients on LPDs with a follow-up equivalent to that of our study (rounded at two years per patient).

If, in the most conservative hypothesis, we calculate that only half of the patients who reached a GFR of ≤10 mL/min would have actually needed dialysis, we would have “saved” 102 years of dialysis, i.e., 5,100,000 Euros, with a net difference of about four million Euros, considering the whole cost of the diet in our center.

If, in the least conservative hypothesis, we calculate that reaching an e-GFR of 15 mL/min or below was equivalent to the start of dialysis, we would have “saved” 385 years of dialysis, with a difference of over 18 million Euros.

Consequently, normalizing our figures, treating 100 patients with an LPD would allow “saving” from 23 to 85 dialysis years, with a minimal difference of at least one million Euros.

## 4. Discussion

The discussion on the potential implications of our study starts from a critical appraisal of a “diet system”, developed in an outpatient unit in which moderately restricted LPDs are proposed to all patients affected by severe and/or progressive CKD, and who have no contraindications, first of all malnutrition.

In this setting, each patient could choose between various diet options and change them over time, and was enrolled into a personalized program of clinical and biochemical controls [21,25]. Such a multiple-choice diet system, focused on tailor-made prescriptions and compliance, makes it possible to analyze a complementary experience as compared to randomized clinical trials, such as the recent one by Garneata or the older one by Brunori [19,20]. While both randomised controlled trials (RCTs) were limited to a subset of the CKD population (10%–30% of cases) and examined/studied severe protein restriction (protein intake: 0.3 g/kg of body weight/day), our study mainly deals with moderate protein restriction (0.6 g/kg/day) and personalized flexible treatments, which allowed us to enroll the vast majority of our CKD patients (over 90% according to a previous analysis) in the diet program, with good compliance and low discontinuation rates [21,25,40].

The main finding of our study is that mortality rates of patients on dialysis-free follow-up were found to be equal to or lower than the USRDS and the Italian and French dialysis registries (Table 2 and Table 3). The advantage was higher in younger patients, and no difference was observed considering the first year of follow-up after the start of dialysis; this observation rules out a carry-over effect of the diet, in keeping with previous observations, and in contrast with the highly debated, long-term report of the Modification of the Diet in Renal Diseases (MDRD) study [16,39,40,41,42].

While our study was not designed to assess the effect on CKD progression, an issue better addressed by RCTs, the potential for delaying the start of dialysis, may be indirectly shown by the patient-years of observation recorded after the first time the e-GFR dropped to or below 15 mL/min, equivalent to “early dialysis start”, or 10 mL/min, equivalent to “late dialysis start” (Table 2 and Table 3, Figure 1). In this “diet or dialysis” phase, the survival advantage versus the USRDS figures remained statistically significant at all ages, while no significant difference emerged with the French or Italian dialysis registries, since the benefit in younger patients (RR of 0.7) did not reach statistical significance (Table 3).

Cost is also an important, growing issue. Considering the patient-years recorded after the e-GFR fell below 15 and 10 mL/min, the savings are remarkable, and range from about one to over four million USD for 100 on-diet patients, the wide range depending on the different figures considered (years of dialysis “saved”: 50% of those recorded below 10 mL/min of e-GFR up to all those recorded below 15 mL/min of e-GFR).

However it is calculated, this data suggests that the economic advantages described for very low-protein diets can also be observed in moderately restricted diets, presumably on a larger scale, considering better patient adherence [43,44,45].

Like all clinical studies, ours has merits and limits.

Besides the limitations shared by non-randomized approaches (the price paid for focusing on personalization rather than standardization), the main limit of our study is that it was carried out in only one center and that despite the fact that the number of subjects it includes is among the highest reported in studies on moderately restricted LPDs, it is still low with respect to the dialysis populations employed for comparison.

Further biases may be present: the drive to continue LPDs as long as possible in patients with short life expectancy, offsetting the survival advantages in older patients; and the fact that having reached a GFR of 10–15 mL/min is not always synonymous with stable kidney failure, thus potentially leading to an overestimation of the dialysis-free follow-up.

The promising results, together with the limits of this first experience, highlight the need for a prospective multicenter analysis including various LPDs and different diet systems and for gathering uniformly defined dialysis cohorts for comparison. In such a setting, other issues, such as the differences between GFR at dialysis start in patients with or without LPDs, not assessed in the present analysis will also be addressed.

The finding that moderately restricted LPDs may enable us to prolong dialysis-free follow-up in large cohorts of CKD patients, allowing survival rates at least comparable to those of dialysis, but at much lower costs, would suggest focusing on implementation of these treatment options in our pre-dialysis networks.

## 5. Conclusions

A flexible and personalized approach to moderately restricted LPDs may allow safely retarding dialysis, as witnessed by the considerable number of years observed on on-diet follow-up after reaching very low GFR levels. The economic advantages are obvious, and the at least equal mortality rates observed in on-diet patients reassure on the safety of this approach. Further large, multicenter studies are needed to validate this approach also in terms of quality of life and to quantify the economic advantages of a wider use of LPDs in the advanced phases of CKD.

## Figures and Tables

**Table 1:** Baseline characteristics of the study population, and of patients who reached 10 mL of GFR, or less.

	All Cases	Pts Who Reached 10 mL of GFR (Data at Start of Diet)
*N*	449	148
Years of observation	847	205 *
Males (%)	276 (61.5%)	88 (59.5%)
Females (%)	173 (38.5%)	60 (40.5%)
Age: median (min–max)	70 (19–97)	68.5 (23–93)
Age over 65 (%)	274 (61%)	82 (55.4%)
Age over 80 (%)	73 (16.3%)	16 (10.8%)
Charlson: median (min–max)	7 (2–13)	6.5 (2–12)
Charlson ≥ 7 (%)	246 (54.8%)	74 (50%)
Charlson ≥ 10 (%)	71 (15.8%)	13 (8.8%)
Diabetes (%)	149 (33.2%)	51 (34.5%)
Cardiopathy (%)	208 (46.3%)	58 (39.2%)
Neoplasia (%)	97 (21.6%)	27 (18.2%)
sCreatinine (mg/dL) median (min–max)	2.8 (0.6–16)	4.0 (1.5–16)
e-GFR-EPI (mL/min) median (min–max)	20 (3–127)	13.8 (3.0–49.5)
Proteinuria (g/day) median (min–max)	0.8 (0.1–11)	1.5 (0.1–11)
Proteinuria ≥ 1 g/day (%)	202 (45.4%)	92 (63.4%)
Glomerulonephritis-systemic disease (%)	95 (21.2%)	38 (25.7%)
Nephroangiosclerosis and/or diabetes (%)	265 (59.0%)	80 (54.1%)
ADPKD (%)	24 (5.3%)	10 (6.8%)

Legend: Charlson: Charlson’s comorbidity Index. E-GFR EPI: GFR according to the CKD-EPI equation. GFR: Glomerular Filtration Rate. EPI: Epidemiology Collaboration. ADPKD: autosomal dominant polycystic kidney disease. * Years of observation after the first recording of an e-GFR at or below 10 mL/min.

**Table 2:** Standardized mortality rates (SMR) analyzed on the basis of the first diet: comparison with United States Renal Data System (USRDS), Italian and French Registries (Reg.) [34,35,36]. All cases.

	Vegan Supplemented	With Protein-Free Food	Other	All Cases
Follow-up/Follow-up one year after discontinuation (years)	375.8	335.7	136.3	847.1
	449.9	347.7	147.2	944.1
Observed deaths: on diet/one year after discontinuation	35	59	6	100
	42	60	7	109
Expected deaths (USRDS): on diet/one year after discontinuation	82.15	105.51	38.31	226.38
	94.52	109.87	40.49	247.30
RR (CI) (USRDS): on diet/one year after discontinuation	0.43 (0.30–0.59)	0.56 (0.43–0.72)	0.16 (0.06–0.34)	0.44 (0.36–0.54)
	0.44 (0.32–0.60)	0.55 (0.42–0.70)	0.17 (0.07–0.36)	0.44 (0.36–0.53)
Expected deaths (Italian Reg.): on diet/one year after discontinuation	50.67	64.55	22.45	137.70
	59.25	67.18	23.60	150.03
RR (CI) (Italian Reg.): on diet/one year after discontinuation	0.69 (0.48–0.96)	0.91 (0.70–1.18)	0.27 (0.10–0.58)	0.73 (0.59–0.88)
	0.71 (0.51–0.96)	0.89 (0.68–1.15)	0.30 (0.12–0.61)	0.73 (0.60–0.88)
Expected deaths (French Reg.): on diet/1 year after discontinuation	48.51	71.70	25.54	143.69
	57.19	74.47	26.96	156.55
RR (CI) (French Reg.): on diet/one year after discontinuation	0.72 (0.50–1.00)	0.82 (0.63–1.06)	0.23 (0.09–0.51)	0.70 (0.57–0.85)
	0.73 (0.53–0.99)	0.81 (0.62–1.04)	0.26 (0.10–0.53)	0.70 (0.57–0.84)
age < 65 years				
Follow-up on diet/one year after discontinuation	189.75	52.42	46.17	287.6
	232.75	54.42	50.17	336.7
Observed deaths: on diet/one year after discontinuation	5/6	3/3	2/2	11/12
Expected deaths (USRDS): on diet/one year after discontinuation	25.78/28.96	9.35/9.71	6.68/7.35	41.72/48.43
RR (CI) (USRDS): on diet/one year after discontinuation	0.19 (0.06–0.45)	0.32 (0.07–0.94)	0.30 (0.04–1.08)	0.26 (0.13–0.47)
	0.21 (0.08/0.45)	0.31 (0.06–0.90)	0.17 (0.03–0.98)	0.25 (0.13–0.43)
Expected deaths (Italian Reg.): on diet/one year after discontinuation	13.94/16.91	4.44/4.62	3.11/3.41	21.48/24.94
RR (CI) (Italian Registry): on diet/one year after discontinuation	0.36(0.12–0.84)	0.68 (0.14–1.97)	0.64 (0.78–2.32)	0.51 (0.26–0.92)
	0.35(0.13–0.77)	0.65 (0.13/1.90)	0.59 (0.71–2.12)	0.48 (0.62–0.94)
Expected deaths (French Reg.): on diet/one year after discontinuation	12.18/14.81	4.82/5.01	3.20/3.54	20.16/23.32
RR (CI) (French Reg.): on diet/one year after discontinuation	0.41 (0.13–0.96)	0.62 (0.13–1.82)	0.63 (0.08–2.26)	0.50 (0.24–0.91)
	0.41 (0.15–0.88)	0.60 (0.12–1.75)	0.57 (0.07–2.04)	0.47 (0.24–0.84)
age ≥ 65 years				
Follow-up on diet/one year after discontinuation	186.0	283.2	90.2	559.6
	216.7	293.3	95.0	604.9
Observed deaths: on diet/one year after discontinuation	30/36	56/57	4/5	89/97
Expected deaths (USRDS): on diet/one year after discontinuation	56.37/65.56	96.58/100.16	31.63/33.14	184.65/198.86
RR (CI) (USRDS): on diet/one year after discontinuation	0.53 (0.36–0.76)	0.58 (0.44–0.75)	0.13 (0.03–032)	0.48 (0.39–0.59)
	0.55 (0.39–0.76)	0.57 (0.43–0.74)	0.15 (0.05–0.35)	0.49 (0.40–0.59)
Expected deaths (Italian Reg.): on diet/1 year after discontinuation	36.73/42.35	60.11/62.56	19.34/20.19	116.22/125.09
RR (CI) (Italian Registry): on diet/one year after discontinuation	0.82 (0.55–1.17)	0.93 (0.70–1.21)	0.21 (0.06–0.53)	0.77 (0.25–0.84)
	0.85 (0.60–1.18)	0.91 (0.69–1.18)	0.25 (0.08–0.58)	0.78 (0.63–0.95)
Expected deaths (French Reg.): on diet/one year after discontinuation	36.34/42.38	66.88/69.46	22.34/23.42	125.56/135.26
RR (CI) (French Reg.): on diet/one year after discontinuation	0.83 (0.56–1.18)	0.84 (0.63–1.09)	0.18 (0.05–0.46)	0.72 (0.56–0.88)
	0.85 (0.56–1.18)	0.82 (0.62–1.06)	0.21 (0.07–0.50)	0.72 (0.59–0.88)

**Table 3:** Standardized mortality rates (SMR), all LPDs together: 240 patients who reached an e-GFR < 15 mL/min and 148 patients who reached an e-GFR < 10 mL/min (follow-up after the first finding of reduced e-GFR).

	e-GFR < 15 (mL/min)	e-GFR < 10 (mL/min)
Follow-up (years)	384.83	204.75
Observed deaths	64	29
Expected deaths (USRDS)	104.43	50.00
RR (CI) (USRDS)	0.61 (0.47–0.78)	0.58 (0.39–0.83)
Expected deaths (Italian Reg.)	63.79	30.16
RR (CI) (Italian Reg.)	1.00 (0.77–1.28)	0.96 (0.64–1.38)
Expected deaths (French Reg.)	67.85	31.51
RR (CI) (French Reg.)	0.94 (0.73–1.21)	0.92 (0.62–1.32)
**<65 years**		
Follow-up (years)	127.83	80.42
Observed deaths	6	4
Expected deaths (USRDS)	18.04	11.65
RR (CI) (USRDS)	0.33 (0.12–0.72)	0.34 (0.09–0.88)
Expected deaths (Italian Reg.)	9.33	6.05
RR (CI) (Italian Reg.)	0.64 (0.24–1.40)	0.66 (0.18–1.69)
Expected deaths (French Reg.)	8.62	5.63
RR (CI) (French Reg.)	0.70 (0.26–1.52)	0.71 (0.62–1.32)
**≥65 years**		
Follow-up (years)	262.00	124.33
Observed deaths	58	25
Expected deaths (USRDS)	86.39	38.35
RR (CI) (USRDS)	0.67 (0.51–0.87)	0.65 (0.42–0.96)
Expected deaths (Italian Reg.)	54.46	24.11
RR (CI) (Italian Reg.)	1.06 (0.81–1.38)	1.04 (0.67–1.53)
Expected deaths (French Reg.)	59.23	25.88
RR (CI) (French Reg.)	0.98 (0.74–1.27)	0.97 (0.63–1.43)
